# Implementation of a surgical unit-based safety programme in African hospitals: a multicentre qualitative study

**DOI:** 10.1186/s13756-019-0541-3

**Published:** 2019-05-30

**Authors:** Lauren Clack, Ursina Willi, Sean Berenholtz, Alexander M. Aiken, Benedetta Allegranzi, Hugo Sax

**Affiliations:** 1 Division of Infectious Diseases and Hospital Epidemiology, University Hospital Zurich, University of Zurich, Rämistrasse 100, HAL 14, 8091 Zurich, Switzerland; 2 Armstrong Institute for Patient Safety and Quality, Johns Hopkins University School of Medicine, Baltimore, USA; 3 Department of Infectious Disease Epidemiology, London School of Hygiene and Tropical Medicine, London, UK; 4 Infection Prevention and Control Global Unit, World Health Organization, Geneva, Switzerland

**Keywords:** Surgical site infection, Infection prevention, Implementation science

## Abstract

**Background:**

A Surgical Unit-based Safety Programme (SUSP) has been shown to improve perioperative prevention practices and to reduce surgical site infections (SSI). It is critical to understand the factors influencing the successful implementation of the SUSP approach in low- and middle-income settings. We undertook a qualitative study to assess viability, and understand facilitators and barriers to implementing the SUSP approach in 5 African hospitals.

**Methods:**

Qualitative study based on interviews with individuals from all hospitals participating in a WHO-coordinated before-after SUSP study. The SUSP intervention consisted of a multimodal strategy including multiple SSI prevention measures combined with an adaptive approach aimed at improving teamwork and safety culture.

**Results:**

Thirteen interviews (5 head surgeons, 3 surgeons, 5 nurses) were conducted with staff from five hospital sites. Identified facilitators included *influential individuals* (intrinsic motivation of local SUSP teams, boundary spanners, multidisciplinary engagement, active leadership support), *peer-to-peer learning* (hospital networking and positive deviance, benchmarking), *implementation fitness* (enabling infrastructures, momentum from previous projects), and *timely feedback* of infection rates and process indicators. Barriers (organisational ‘constipators’, workload, mistrust, turnover) and local solutions to these were also identified.

**Conclusions:**

Participating hospitals benefitted from the SUSP programme structures (e.g. surveillance, hospital networks, formation of multidisciplinary teams) and adaptive tools (e.g. learning from defects, executive rounds guide) to change perceptions around patient safety and improve behaviours to prevent SSI. The combination of technical and adaptive elements represents a promising approach to facilitate the introduction of evidence-based best practices and to improve safety culture through local team engagement in resource-limited settings.

## Background

Healthcare associated infections (HAIs) represent one of the most frequent adverse events affecting patient safety worldwide [1]. Surgical site infections (SSI) are the most surveyed and most frequent type of HAI in low- and middle-income countries (LMICs) as well as Europe and the United States (US) [2–6]. In the recently published African Surgical Outcomes Study, infection was the most frequent postoperative complication, affecting 10.2% of patients [6]. By contrast, SSI rates in high-income countries vary between 1.2 and 5.2% [3].

In light of the global burden of SSI, their reduction is a priority [7]. Specifically, a Surgical Unit-based Safety Programme (SUSP) has been developed by the American Agency for Healthcare Research and Quality to reduce SSI and other surgical complications [8]. The SUSP approach is based on the use of both technical and adaptive strategies and tools to enhance local safety culture and empower front-line care providers to address preventable harm [9]. Implementation of similar multifaceted approaches have been associated with significant and sustained reductions in HAIs and improvements in safety culture in high-income countries [9–13]. A substantial knowledge gap exists, however, regarding implementation of strategies to reduce SSIs in LMICs, and sub-Saharan Africa in particular [14]. It is well recognised that contextual factors, such as local patient safety culture, play a role in the successful implementation of evidence-based practices [15–17]. Accordingly, the successful implementation of specific improvement approaches in US hospitals alone does not guarantee its success in other settings [18]. To test the validity of the SUSP approach in other settings, the World Health Organisation (WHO) Service Delivery and Safety in collaboration with Johns Hopkins Medicine Armstrong Institute for Patient Safety and Quality have, beginning in 2013, partnered with experts and frontline providers in a group of African hospitals to adapt and test the impact of the SUSP approach through a multicentre before-after cohort study in five sub-Saharan African hospitals [19].

We performed an interview-based, qualitative study in all five African hospitals participating in the aforementioned SUSP Study [19] to draw meaning from their experiences and inform how the SUSP programme may best be adapted for use in additional settings. This research was guided by two primary study questions: What are the facilitators and barriers to implementation of a comprehensive unit-based safety programme to reduce surgical site infections in these five African hospitals? What influence, if any, did SUSP have on the safety culture in participating hospitals?

## Methods

### The SUSP study intervention

The multimodal SUSP intervention in five sub-Saharan African hospitals (study hospitals) combined *technical elements* (six technical SSI prevention measures to be implemented or improved) and *adaptive elements* (implementation and monitoring tools) to enhance patient safety culture by changing the attitudes, values and beliefs of people who deliver care (Table 1) [8]. Prior to implementation, the WHO and Johns Hopkins Armstrong Institute for Patient Safety and Quality worked with senior surgeons (surgical team leaders) from study hospitals to adapt and co-develop the SUSP tools and protocols. A small budget was provided to each hospital to be used only for costs incurred from data collection extending beyond normal clinical services. These funds were not used for procurement of equipment or products related to the project or for remuneration of pre-existing staff. Local core SUSP teams were established at each participating study hospital. These local teams were composed of a surgical team leader as well as nurse and surgeon “champions”, defined as individuals responsible for advocating local implementation of the SUSP intervention among their colleagues. Local teams were entirely responsible for adaptation and implementation of the SUSP intervention, with staff from WHO forming a central coordinating team and providing technical expertise on project management and data collection [19].

**Table 1:** Intervention implementation activities

Technical SSI Preventative Measures^1^	Adaptive Elements^2^
• Preoperative patient bathing • Avoiding hair removal or performing it with clippers • Optimisation of surgical site skin preparation • Optimisation of surgical hand preparation • Optimisation of surgical antibiotic prophylaxis (timing, dose, type of antibiotic, re-dosing) • Improving discipline in the operating room (limiting number of people and door opening during operation)	• Formation of local SUSP perioperative team • Hospital survey on patient safety, to raise awareness and assess current status of patient safety culture • Patient safety video played by local surgical leaders • CUSP adaptive tools: o Staff safety assessment o Learning from defects • Morbidity and mortality meetings • Participation in monthly multisite SUSP webinars • Conduct of local educational meetings • Feedback of data on SSI surveillance and compliance with SSI preventive measures

SSI = survival site infection. SUSP=Surgical Unit-Based Safety Programme. CUSP=Comprehensive Unit-Based Safety Programme [1].Support materials related to the technical SSI preventive measures are available at https://www.who.int/infection-prevention/countries/surgical/en/ [2]. Materials from the CUSP study used in this project are available at https://www.ahrq.gov/professionals/quality-patient-safety/hais/tools/surgery/index.html

### Participants

All five African hospitals included in the SUSP intervention study [19] were also included in the qualitative study (Table 2). We conducted semi-structured interviews with 2–3 individuals from each hospital who were closely involved in implementation activities and therefore purposefully selected to provide the richest description of implementation experiences. These interviewee participants were members of the local core SUSP teams from each hospital, e.g. the surgical team leaders, surgeon champions, and nurse champions.

**Table 2:** Characteristics of participating hospitals

Country	Type	Setting
Kenya	Private, mission hospital, 360 beds	Rural
Uganda	Public sector, tertiary referral, 1500 beds	Urban
Uganda	Private, mission hospital, 260 beds	Rural
Zambia	Public sector, tertiary referral, 851 beds	Urban
Zimbabwe	Public sector, tertiary referral, 1500 beds	Urban

### Data collection procedure

Interviews were conducted during a three-day meeting at the conclusion of the project by two independent researchers (LC, VG) who had not been involved in the SUSP project implementation. All interviews were audio-recorded, lasted approximately one hour and followed a semi-structured interview guide, which addressed individual SUSP involvement, the SSI prevention intervention, key individuals and leadership support, data collection and sharing, patient safety culture, and the overall SUSP experience (Table 3). All audio-recordings were transcribed verbatim and included in the analysis.

**Table 3:** Semi-structured interview guide

SUSP Involvement	
How did you first become involved in the project?	
When you first learned about the project, what were your expectations?	
Who decided that your hospital participates and why?	
SSI Prevention intervention	
Think about your SSI prevention intervention. Can you walk me through the intervention effort?	
What elements of your SSI prevention intervention did you perceive as most useful in your setting?	
What elements of your SSI prevention intervention were most difficult to implement, if any?	
Key individuals/Leadership support	
Who was involved in your SSI intervention team?	
Who would you say was most supportive to the SUSP project?	
In what way, if at all, did leadership support or hinder the intervention?	
Data Review and Sharing	
How did people in the hospital hear about the results of the SUSP effort, if at all?	
What problems, if any, have you had with getting the data you needed?	
Safety Culture	
What did you think of the HSOPS survey questions?	
Have you noticed any change in the patient safety culture in your institution?	
Aside from this project, do you have any other patient safety programs in your hospital?	
Your SUSP experience (wrap-up)	
What do you think the SUSP experience represented for your unit/facility?	
What do you think the SUSP experience represented for you personally as a health professional?	

**Abbreviations:** SUSP=Surgical Unit-Based Safety Programme. SSI = surgical site infection. HSOPS=Hospital survey on patient safety

### Ethics approval

Participation was voluntary and participants were free to withdraw from the interview at any time. All interviewees provided written informed consent. This qualitative study was considered by the WHO Ethics Review Committee (ERC) as exempted from ERC review. The SUSP intervention was approved by ERC (RPC632, WHO HQ ERC) and the institutional ethics committees of all five participating hospitals. To ensure confidentiality of our participants, we report our qualitative findings in aggregate form that does not identify hospitals or individuals.

### Analysis

Data collection and analysis followed an iterative approach whereby preliminary review of data influenced ongoing data collection. When data collection was concluded, an inductive analysis was conducted whereby two researchers (LC, UW) began by reading interview transcripts and establishing a codebook. Both researchers then independently read and coded all transcripts, meeting periodically to discuss the coded transcripts, adjust the codebook, and settle any coding discrepancies through consensus discussions. Thematic analyses were first conducted at the hospital site level to better understand the dynamics of the implementation process at each hospital. Finally, cross-case analyses were conducted using structured matrices to identify how barriers and facilitators were manifest across sites [20].

To validate credibility of our findings, we performed ‘member checking’ [21] by sharing our preliminary interpretations with interview participants. This process consisted of a structured presentation of our findings followed by group discussion with local core SUSP teams from all five hospitals. Participants were invited to provide feedback on our initial interpretations and to add or modify our accounts to ensure that they accurately reflected their reality.

### Sensitising framework

This inquiry was informed theoretically by the Consolidated Framework for Implementation Research (CFIR), a collection of constructs within five domains (intervention characteristics, outer setting, inner setting, characteristics of individuals involved, and implementation process) which have been shown to influence implementation effectiveness [22]. This framework served as a sensitising scheme [23], particularly for establishing the interview guide and orienting the inductive analysis; however, our data collection was designed to capture a broad range of experiences, not limited to those addressed by this framework.

## Results

A total of 13 interviews were conducted across five hospital sites. Interviewees included team leaders (*n* = 5, 39%), surgeon champions (*n* = 3, 23%), and nurse champions (n = 5, 39%). None of the invited interviewees declined to participate. We identified several facilitators and barriers that were critical to the implementation process (Table 4). These facilitators, barriers, and local solutions identified to overcome barriers, are presented in the following sections. Finally, the impact on local safety culture is discussed.

**Table 4:** Identified facilitators and barriers

Facilitators		
Influential individuals	Characteristics of individuals in local core SUSP team	The personal commitment and motivation of the individuals primarily responsible for implementing the SUSP intervention
	Boundary spanners	The involvement of individuals who have multiple roles and traverse institutional boundaries to accelerate change and gain broad stakeholder engagement
	Active leadership support	Individuals in leadership positions demonstrating their support for the project through their physical presence and making resources available
Peer-to-peer learning across participating hospitals	Hospital networking and positive deviance	Opportunities throughout the project to exchange in-person or virtually with individuals from other hospitals and learn from examples of positive deviance
Implementation fitness	Momentum	Building on the energy that was already channelled towards quality improvement thanks to previous projects
	Enabling infrastructures	Benefitting from knowledge, surveillance systems, and practices already in place because of previous quality improvement initiatives was a foundation to support the introduction of new practices
Timely feedback	Tension for change	The feedback of sub-optimal baseline data showing room for improvement to create a sense of urgency to change practice
	Ongoing feedback	Regular feedback of infection rates and process indicators throughout the project to sustain engagement
Barriers		
	Organisational constipators	Lack of leadership support that stifles the efforts of other individuals to effect change
	Workload	The significant SUSP workload was often taken on by SUSP core team members in addition to their pre-existing duties
	Mistrust	Lack of financial incentives in the current project, as had come to be expected in previous projects, led to scepticism
	Staff turnover	The rapid turnover of surgical staff created difficulties to keep everyone trained on SUSP protocols

SUSP=Surgical Unit-Based Safety Programme

### Facilitators

#### Influential individuals

Individuals played a central role in facilitating implementation of the SUSP interventions throughout the implementation process: passionate and highly-motivated individuals were identified to be part of local core SUSP teams and drive local change, boundary spanning individuals helped to break down organisational restrictions and accelerate change across their organisation, and individual members of hospital leadership facilitated purchasing of necessary materials and infrastructures.

##### Local core SUSP teams

The establishment of local, multidisciplinary core SUSP teams composed of “passionate”, “courageous”, and “intrinsically motivated” individuals was cited as a central facilitator to implementation of the SUSP interventions. These individuals were the driving force of the SUSP interventions in their respective hospitals and they channelled their intrinsic motivation and perseverance to overcome challenges faced during the SUSP implementation.

A nurse champion explained, “*You need to be courageous. Actually when you are implementing something, you have to put your foot down. It's not easy.*”

##### Boundary spanners

Boundary spanners, defined as individuals who have multiple roles and traverse institutional boundaries [24], facilitated implementation by accelerating change across departments and establishing buy-in with multiple stakeholders. Several of the SUSP surgical team leaders were also members of their hospital leadership. Such individuals enabled hierarchical boundary spanning and particularly facilitated the purchase of materials, such as sterile drapes and chlorhexidine antiseptic, and installation of infrastructures, such as showers, necessary for the SUSP project.

One boundary spanning team leader described, *“I got the senior management team, because I was seated in the same room, to agree that this was good for the hospital, good for our patients and that it was cost effective.*”

Local SUSP team members also actively spanned boundaries by seeking input from additional stakeholders, such as pharmacists, cleaning staff, theatre staff, and other surgeons. This helped establish broad commitment to the project and brought to attention areas for improvement that hadn’t previously been considered.

According to a surgeon champion, “[if] *you want to change the antibiotic use, you get the pharmacists involved because those are the owners of the antibiotics. …then it's not like you're telling the pharmacist what to do, but the pharmacist is saying, ‘okay, this is what we're going to be doing’.”*

##### Active leadership support

Members of hospital leadership who demonstrated their active support, for example through participation in executive rounds and facilitating the purchase of materials necessary for the intervention, were facilitators. The adaptive SUSP tool for leading executive rounds [25], which involved a senior executive walk-through of the clinical area led by a frontline clinician, was further cited as a facilitator to engaging hospital leadership to gain their support.

A surgeon champion explained, *“[our director] would come [on the executive rounds] and there are places in the hospital he had never set foot. …he would just be shaking his head saying, ‘well, I didn't know this happens in this hospital.’ …and we would get results almost instantly.”*

A nurse champion described, “*when we were doing executive rounds, we found that nurses were using containers to bathe patients and there were no showers….within 24 hours [of the executive round], everything was cleared, there were instant showers*.”

In contrast, a lack of concrete leadership support was cited as a major barrier to SUSP implementation, as later discussed under ‘Barriers’.

#### Peer-to-peer learning across participating hospitals

Throughout their participation in the SUSP programme, hospitals had several opportunities to meet and interact, both in person and virtually, with individuals from other participating hospitals during networking events and virtual webinars.

##### Networking events

The multiple networking events throughout the project created opportunities for core SUSP team members to exchange about their experiences and enabled “positive deviance” [26], whereby hospitals could learn from and emulate the success of their peers.

A nurse champion described, “*[the kick-off meeting was] an eye-opener for us. Because you meet with different people from different sites, you hear how they do their way, we share our experiences*.”

A team leader shared, *“we had a meeting with [head surgeon from another SUSP hospital]. They had already launched, so he was good support.”*

##### Virtual webinars

The monthly webinars (internet-based meetings and presentations) also created friendly competition between hospitals and an opportunity to discuss and identify solutions to the challenges they were facing. This exchange forum also helped participating hospitals to establish synergies, for example purchasing alcohol-based hand rub from the same producer to reduce costs.

A team leader said, “s*o we sit and we just say, ‘Guys, this is how well we’ve done. We did a webinar and we are behind all the other hospitals. Don’t let [neighbouring hospital] beat us!”*

Another team leader described, “*when we had an alcohol-based hand rub challenge, we communicated with [another hospital] and discovered that well they have the same problem. We agreed that now we all get alcohol from the same place, which is even cheaper and can be [purchased] in bigger quantities*.”

#### Implementation fitness

Implementation fitness, an institution’s ability to integrate evidence-based recommendations into practice, was strengthened in some hospitals that had previously participated in quality improvement initiatives and had therefore already established momentum and enabling infrastructures.

##### Momentum

Interviewees described that participation in SUSP helped them to “*maintain the momentum*” that had been established through participation in previous initiatives. For example, two hospitals had been involved in the African Partnership for Patient Safety (APPS) project that had been initiated three years prior, which aimed to support patient safety improvements through hospital-to-hospital partnerships [27].

One team leader recalled, “*During APPS there was a lot of Science of Patient Safety training. Of course there was so much resistance. Many things were really big shifts for us. Then [SUSP] was the best thing for us because at that time we were just closing the project with APPS… so we thought this was going to help us to also maintain the general awareness around patient safety.”*

##### Enabling infrastructures

Interviewees further shared that experience with previous research and quality improvement initiatives meant they had already established ‘enabling infrastructures’, such as surveillance and patient follow-up systems and surgical checklists, which facilitated SUSP implementation.

A nurse champion described, “*I have also worked in other international research projects. So, even when it came to surveillance and follow-up of patients on the SUSP project, it was not hard because it was something I had done before*.”

#### Timely feedback

Regular feedback of data on SSI surveillance and compliance with SSI preventive measures was an important facilitator, particularly to create a tension for change at the beginning of the project and to sustain engagement through ongoing feedback throughout the project.

##### Tension for change

During the intervention kick-off meeting, feedback of sub-optimal baseline data on SSI rates was a catalyst, creating tension for change and a sense of urgency among colleagues.

A nurse champion described, “*so that’s how our intervention began…we shared with them the results of the baseline phase, and man they said, ‘“Eh, we are doing poorly. What do we do then?” They got concerned. They got into the thing!*”

##### Ongoing feedback

Regular feedback of results, both SSI rates and compliance with SSI preventive measures, throughout the implementation helped to demonstrate the effectiveness of actions taken and to sustain engagement in the project. Such ongoing feedback was especially critical to establishing confidence in components of the SUSP intervention, such as limiting post-operative antibiotics, of which colleagues were sceptical of the safety.

A surgeon champion shared, “*We had another session about three months into the intervention and we showed that the number of patients who got post-operative infections was reducing. At the same time the number of patients who were getting post-operative antibiotics was also reducing. I think that was in a way enough to convince people*.”

### Barriers

#### Organisational constipators

Whereas positive leadership was a facilitator, a lack of active leadership support was a clear barrier, particularly when hospital leadership claimed to be supportive of the programme, but failed to take concrete actions to demonstrate their support. Such individuals have been termed organisational ‘constipators’ [28].

One team leader described, “*I think [the hospital director] was already on board, but they are pulled apart by too many demands on their time… We just couldn’t get them to be more involved.*”

This became a concrete barrier to implementation, as described by the team leader, “*whereas the people on the ground seem to be willing to do more, or have done a lot, I think they have reached a point where they would need more help from people above them to try and get more done*.”

The lack of leadership support in this scenario effectively stifled, or ‘constipated’, the efforts already made by other team members.

##### Local solution

Interviewees mentioned that adaptive tools to guide executive rounds helped overcome the challenge of getting executives on the wards. Executive rounds helped to make leadership acutely aware of on-the-ground challenges and motivate them to support SUSP implementation.

A surgeon champion described, “*having something formal and something to follow is helpful*. *I think if it just went without a guided tool or anything, I’m sure [it] would have made too much noise.”*

#### Workload

Implementation of SUSP activities involved an increased workload, particularly for the core SUSP team, which was a barrier to implementation. Individuals in the SUSP team often took on this role in addition to their pre-existing duties.

A nurse champion explained, “*It's a lot of work. And then there are so many meetings, you have to sacrifice a lot. At the same time, you're not expected to take time off from your normal duties.”*

##### Local solution

To manage the additional workload, members of the core SUSP team often sought the assistance of other staff members, to whom they delegated activities such as data collection or creating educational posters. The delegation of activities to other staff members had the joint advantage of building a greater base of individuals involved in and committed to the SUSP project.

A surgeon champion said, *“I did a lot of delegation to the rest of the team to just, everybody to do their own bit.”*

#### Mistrust

Some SUSP core teams experienced difficulty building the trust of staff in their hospitals – particularly regarding the repartition of funds that were provided to each participating hospital. Although these funds were not used for remuneration of pre-existing staff, some colleagues nonetheless suspected that the SUSP core team was receiving financial incentives. Colleagues with such suspicions were sometimes unwilling to participate in the project and this created “distance” between the SUSP core team and the hospital staff.

One team leader described, “*I think there’s a general [perception] that most research projects have a lot of money… If [my colleagues] don’t see obvious money reaching them as well, they feel you are holding and keeping it to yourself*.”

This mistrust was further exacerbated by previous projects that had provided financial incentives in exchange for individual participation, for example returning forms.

A nurse champion described, “*As it has been happening, whenever there is a project, there are some incentives. So for this one, wherever I told them that there's literally nothing, they couldn't believe it.”*

Such financial incentives provided in previous studies were misleading – causing staff to expect monetary rewards for their efforts and thus to mistrust projects without financial rewards.

##### Local solution

Instead of financial incentives, some SUSP hospitals expressed appreciation in the form of non-financial incentives and symbols of appreciation, such as breakfast and snacks during SUSP presentations to motivate their staff and incite them to attend.

A team leader described, *“just something small. Like breakfast, tea, snacks... To show you value them and their time.”*

#### Staff turnover

High staff turnover was frequently cited as a barrier to implementation of the SUSP interventions – particularly to keeping new staff trained on SUSP protocols and the science of patient safety.

A nurse champion described, “*We have a high turnover in our hospital, especially the nurses. They are always looking for greener pastures. So, every time you educate this group about the science of safety, do not shave, how you're supposed to do the bathing, then after that, they are gone*.”

##### Local solution

In light of frequent turnover, multiple hospitals implemented continuous education of newly arriving staff to maintain awareness about the SUSP programme and protocols.

A team leader said, *“now we’re trying to get a policy whereby the newly recruited staff is given training on patient safety.”*

### Patient safety culture

Our qualitative study also aimed to identify what impact, if any, SUSP had on local patient safety culture in participating hospitals. When reflecting on patient safety in their hospitals, interviewees frequently mentioned that patient safety had not been widely thematised prior to the SUSP interventions and that, with some exceptions, few specific actions had previously been undertaken to improve local patient safety culture. Interviewees further recalled specific challenges to promoting patient safety, such as ambivalence about adverse events and a culture where individuals were blamed and seen as the source of adverse events.

A surgeon champion described, “*unfortunately, where we work, [patient safety] is not something that's emphasised… any adverse events are just looked at like – it's just something that happens. No one really tries to look back and think, what could we have done to make a difference in this.”*

Another surgeon champion described, “*the mentality is always if something’s wrong, then there’s someone to blame.”*

Following the implementation of SUSP, however, interviewees described an improvement in certain aspects of patient safety culture, which they described as being a direct result of the adaptive SUSP elements. For example, several hospitals established morbidity and mortality meetings as an occasion to reflect on the system-level causes of adverse events. During these meetings, the adaptive tools, such as “Learning from Defects” [29], were cited as useful tools to shift blame away from individuals and to focus on the system-routed issues to be addressed. In general, a learning curve was observed, whereby increasing awareness about patient safety issues resulted in a gradual shift in patient safety culture. Despite the felt progress, interviewees still noted room for improvement in areas such as speaking up, and not assigning blame.

Perhaps one of the “*greatest successes*”, as described by a surgeon champion, was, “*getting people to think about patient safety. …[whereas previously] at the very remote back of your mind you would think about it but not really have a way of bringing it out but at least now there is a way of bringing it out. For me that's the greatest success is the awareness that patient safety is an issue and there's something that can be done about it to just improve patient safety.”*

## Discussion

This qualitative study draws on the experience of five African hospitals and reveals the following key facilitators to implementation of the SUSP programme: *influential individuals*, who spanned boundaries to accelerate change and establish support for the intervention among colleagues and hospital leadership; *peer-to-peer learning across participating hospitals*, which fostered synergies and positive deviance across institutions; *implementation fitness*, where hospitals built on the momentum and enabling infrastructures that had been established through previous quality improvement initiatives; and *timely feedback* of data, which established tension for change among participants and sustained project engagement. Whereas barriers such as *organisational constipators*, *workload*, *mistrust* and *staff turnover* challenged implementation in some hospitals, much can also be learned from the locally developed solutions. These included using the SUSP adaptive tools to engage reticent hospital leaders, delegating SUSP tasks to distribute workload, avoiding financial incentives in favour of symbolic gestures of appreciation, and continuous staff training.

Our findings support the conclusions of previous and recent systematic reviews also highlighting the importance of leadership support, multidisciplinary engagement, ongoing staff training and performance evaluation and feedback as critical components of effective implementation of surgical checklists and SSI prevention interventions [30–32]. Specifically, a 2015 review and thematic synthesis of qualitative evidence by Bergs et al., in which 2 of 18 included studies reported data from LMICs, found that “executive leadership”, “organisational culture” and “communication and teamwork” were the most frequently reported elements of local context that influenced implementation of surgical checklists [30]. The authors concluded that implementation of surgical checklists requires more than eliminating isolated barriers and supporting facilitators and that, instead, implementation leaders should foster shared motivation and work together with staff to adapt existing routines. These conclusions are supported by our findings and further justify the comprehensive SUSP approach, which combines technical and adaptive elements to facilitate the introduction of evidence-based best practices while working to improve safety culture.

Our findings about the important role of hospital networks builds on the findings of Dixon-Woods and others, who found that establishing quality improvement networks with “strong horizontal links” across hospitals supported implementation by exerting normative pressure on members [24, 33]. We additionally found that participation in the SUSP network supported implementation by enabling peer-to-peer learning and supporting the identification of synergies across hospitals. A study by Aveling and colleagues comparing implementation of the safe surgery checklist in high- and low-income countries revealed that African hospitals faced more challenges to implement the checklist than sites in the United Kingdom because they lacked features that supported the implementation, such as pre-existing data collection and audit systems [34]. This is consistent with our observation that implementation fitness thanks to previous participation in quality improvement initiatives helps to establish enabling infrastructures that facilitate the implementation of best practices. Finally, our results are consistent with Mathauer and Imhoff, who found financial incentives were limited in their effectiveness and sustainability for increasing motivation of health professionals in Benin and Kenya, thereby supporting the conclusion that financial incentives should be avoided and non-financial gestures of appreciation or other measures to promote intrinsic motivation should be favoured as part of a holistic quality management model [35].

Similar to Ente and colleagues, who found that 75% of healthcare professionals surveyed in Western Nigeria and Northern Uganda attributed adverse events to individual mistakes [36], interviewees in our study described a baseline culture of individual blame and ambivalence, where adverse events were not regularly discussed, much less examined to identify learning opportunities. The major improvement in safety culture was thus having the tools and the language necessary to identify and learn from patient safety issues that would have formerly been dismissed.

Our study findings should be interpreted in light of some limitations. This qualitative inquiry was conducted at the conclusion of the intervention period; it is possible that interviewees’ recollection of certain events could be influenced by subsequent events or by interpretation of study outcomes. Due to the nature of data collection, interviews were limited to individuals who were actively involved in planning and implementation of the SUSP intervention in their respective hospitals. Further interviews with other project stakeholders may have revealed additional insights.

A strength of this study is that this research was led by a team of researchers purposefully not having been involved in SUSP implementation so as not to bias our findings. The different cultural background of the researchers further justified the use of member checking, in which we shared our preliminary findings with research participants to ensure their credibility from the perspectives of those who live and work in the studied context [37].

## Conclusions

We conclude that a complex combination of local contextual factors, rather than isolated barriers and facilitators, influenced implementation in these 5 African hospitals. Namely, leveraging the intrinsic motivation of clinical leaders was key to SUSP implementation in these five hospitals. These individuals spanned organisational boundaries to establish broad support for the SUSP programme, building on enabling infrastructures (e.g. surveillance, hospital networks, formation of multidisciplinary teams) and adaptive tools (e.g. learning from defects, executive rounds guide) to change perceptions and behaviours to prevent SSI. Availability of qualitative information on factors influencing implementation and uptake of IPC interventions in settings with limited resources is scarce and thus, the lessons learned in these hospitals are critical for the future adaption of the SUSP project and for other quality improvement work in other resource-limited settings. The combination of technical and adaptive elements represents a promising approach to facilitate the introduction of evidence-based best practices and to improve safety culture through local team engagement in resource-limited settings. This is also useful to inspire global strategies for SSI prevention implementation.

## Abbreviations

CFIR: Consolidated Framework for Implementation Research
ERC: Ethics Review Committee
HAI: Healthcare associated infection
LMIC: Low- and middle- income country
SSI: Surgical site infection
SUSP: Surgical unit-based safety programme
US: United States
WHO: World Health Organisation
